# Test–retest reliability of Kinect’s measurements for the evaluation of upper body recovery of stroke patients

**DOI:** 10.1186/s12938-015-0070-0

**Published:** 2015-08-04

**Authors:** A Mobini, S Behzadipour, M Saadat

**Affiliations:** Mechanical Engineering Department, Sharif University of Technology, Tehran, Iran

**Keywords:** Reliability, Kinect, Stroke, Motor assessment, Neurorehabilitation

## Abstract

**Background:**

Performance indices provide quantitative measures for the quality of motion, and therefore, assist in analyzing and monitoring patients’ progress. Measurement of performance indices requires costly devices, such as motion capture systems. Recent developments of sensors for game controllers, such as Microsoft Kinect, have motivated many researchers to develop affordable systems for performance measurement applicable to home and clinical care. In this work, the capability of Kinect in finding motion performance indices was assessed by analyzing intra-session and inter-session test–retest reliability.

**Method:**

Eighteen stroke patients and twelve healthy subjects participated in this investigation. The intra-session and inter-session reliability of eight performance indices, namely mean velocity (*MV*), normalized mean speed (*NMS*), normalized speed peaks (*NSP*), logarithm of dimensionless jerk (*LJ*), curvature (*C*), spectral arc length (*SAL*), shoulder angle (*SA*), and elbow angle (*EA*), were assessed using intra-class correlation coefficient (*ICC*), standard error of measurement (*SEM*) and coefficient of variation (*CV*).

**Results:**

The results showed that, among the performance indices, *MV*, *LJ*, C, SA and *EA* have more than 0.9 *ICC* together with an acceptable *SEM* and *CV* in both stroke patients and healthy subjects. Comparing the results of different therapy sessions showed that *MV*, *LJ* and C are more sensitive than other indices, and hence, more capable of reflecting the progress of a patient during the rehabilitation process.

**Conclusion:**

The results of this study shows acceptable reliability and sensitivity across the sessions for *MV*, *LJ* and *C* measured by Kinect for both healthy subjects and stroke patients. The results are promising for the development of home-based rehabilitation systems, which can analyze patient’s movements using Kinect as an affordable motion capture sensor.

## Background

Using virtual reality systems for stroke rehabilitation is a flourishing field in physical and neurological rehabilitation. Such systems can help patients have a more intensive and entertaining training. They are commonly composed of a sensory device to capture the patient’s movements, and a computer interface to communicate with the patient and guide him through the intended tasks. Various sensors have been used in the development of such systems, including very simple ones, such as IMUs (inertial measurement units), and more sophisticated ones, such as sensorized robotic arms. The potentials of such systems in physical and neurological rehabilitation have been investigated by various researchers. For instance, Tsoupikova et al. [1] and Wade et al. [2] have proven the capability of virtual reality systems in the hand rehabilitation of post stroke patients. Deutsch et al. [3] have investigated the efficacy of Nintendo Wii in the rehabilitation of cerebral palsy patients. Moreover, Medrum et al. [4] have studied the applicability of Nintendo Wii in the balance rehabilitation of neurologic patients. Patel et al. [5] have reviewed the utilization of wearable sensors in rehabilitation. Also, Bao et al. [6] have discussed the role Kinect can play in the recovery of upper limbs of stroke patients.

High cost of such systems, which is mostly due to the sensor hardware, is one of the main challenges. As a result, using modern motion sensing game controllers such as Microsoft Kinect [7], Nintendo Wii remote, and Balance Board [8] has been widely investigated. These new sensors have much lower cost than the clinical ones, namely Vicon [9] and Optotrak [10], because they are simpler, less accurate, and are being mass produced in over 20 million numbers. Despite being recently introduced to the market, these new motion sensors have been used in various commercial rehabilitation applications such as Wiifit [11], SeeMe [12], Virtualrehab [13], and JINTRONIX [14].

In several previous works, it has been shown that stroke patients’ current state and amount of recovery can be assessed based on their hand movement quality. Rohrer et al. [15] have assessed movement smoothness changes during the recovery of stroke patients. Van Dokkum et al. [16] have investigated the contributions of kinematics in the assessment of upper limb motor recovery of stroke patients. Osu et al. [17] have studied the quality of hand movements using three dimensional curvature. Moreover, Balasubramanian et al. [18] have defined a new metric for quantifying the smoothness of hand movements of stroke patients. Hogan et al. [19] have discussed sensitivity of jerk smoothness measure in determining hand movement performance. These quality measures, which mostly include kinematic indices of motion such as mean velocity, dimensionless jerk, motion curvature, etc., can be computed automatically during therapy sessions in order to provide in-depth information about the patient’s impairments and recovery process. Determining these features by using clinical measurement systems such as Xsens [20], Optotrak [10] and Vicon [9] is very expensive and can be applied only in labs equipped for this purpose. On the other hand, using game controllers, despite being more affordable and available in almost any environment, has drawbacks such as lower accuracy, precision, and, more importantly, the reliability of outputs.

In the related researches, validity and reliability of Wii balance board using Nintendo Wii Fit balance scores [21], and balance scores measured by a balance assessment software have been discussed [22]. Wii remote controller’s accuracy, validity and reliability for measuring head posture [23] has also been determined. Kinect’s ability in pose estimation [24] and measuring joints positions [25] has been investigated in the literature as well. Also, the reliability and accuracy of measuring the range of motion [26], joints positions [27], and joints angles [28] using Microsoft Kinect has been studied by various researchers.

Although many studies have been carried out on the accuracy and reliability of these sensors, the measurement targets have been the raw data which is directly measured by the sensors. Measuring performance indices and the corresponding measurement reliability, which is essential for application of such sensors in rehabilitation, have been mostly overlooked. The study of Elgendi et al. [29], in which they calculated speed and classified subjects using Kinect, is the closest one to using Kinect for measuring movement performance indices. However, even in this work the reliability of such measurements is not determined.

In order to fill this gap, this study attempts to assess the test–retest reliability of Kinect’s measurements of performance induces for the evaluation of upper body recovery in stroke patients. Among various methods available for determining test–retest reliability, this work uses the intra-session and inter-session approach. Movement performance indices were measured on 30 subjects using Kinect and the measurement reliability was analyzed. Furthermore, the sensitivity of the performance indices measured by Kinect was investigated.

## Methods

### Subjects

A group of 18 stroke patients (8 females and 10 males; aged 50 ± 16) and 12 healthy subjects (4 females and 8 males; aged 46 ± 15) participated in this research. The tests were taken at the Red Crescent Society Rehabilitation Center (Tehran, Iran). Eleven of the patients were in the sub-acute phase (less than 6 months had passed from their unilateral cerebrovascular accident or CVA), and seven of them were at the chronic phase (more than 6 months had passed from their unilateral CVA). The inclusion criteria consisted of the existence of a single unilateral CVA and the occurrence of movements with more than 15 degrees in the impaired shoulder and elbow. However, any sever visual impairments, apraxia, or neglect syndromes lead to the exclusion of subjects from the tests. All subjects were informed about the study and willingly participated in the tests. Their consents were approved by the local scientific and ethics committees.

### Data capture program

The subjects’ hand movements were measured by the Microsoft Kinect for Xbox 360 using Microsoft Kinect’s skeleton tracking driver version 1.7 [7]. A program was designed for this purpose using C# and Microsoft XNA game studio [30]. During the test, subjects sat or stood in front of a video screen running a graphical interface program. Kinect’s distance from each subject was about 2.7 m. This was set so that Kinect could see the full body of the subject. As the sitting option was used in the skeleton tracking program and only upper body joints were tracked, there was no difference between tracking subjects in standing or sitting positions.

**Fig. 1:**
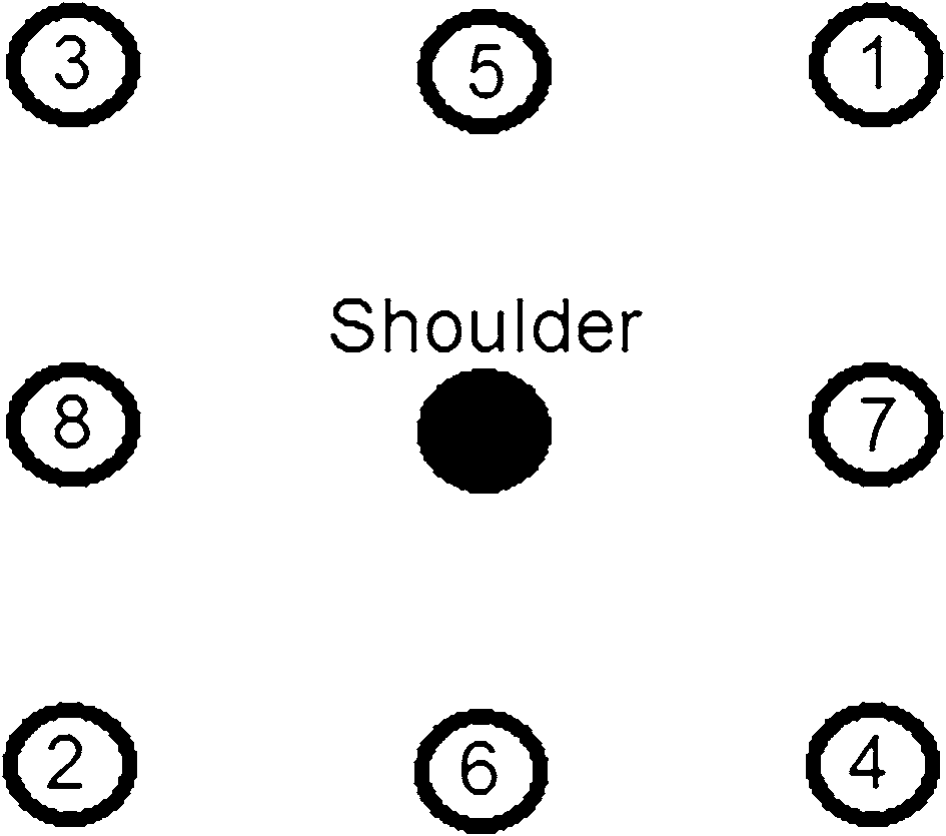
Target pattern for the assigned movement task. All targets are in a plane parallel to the frontal plane.

**Fig. 2:**
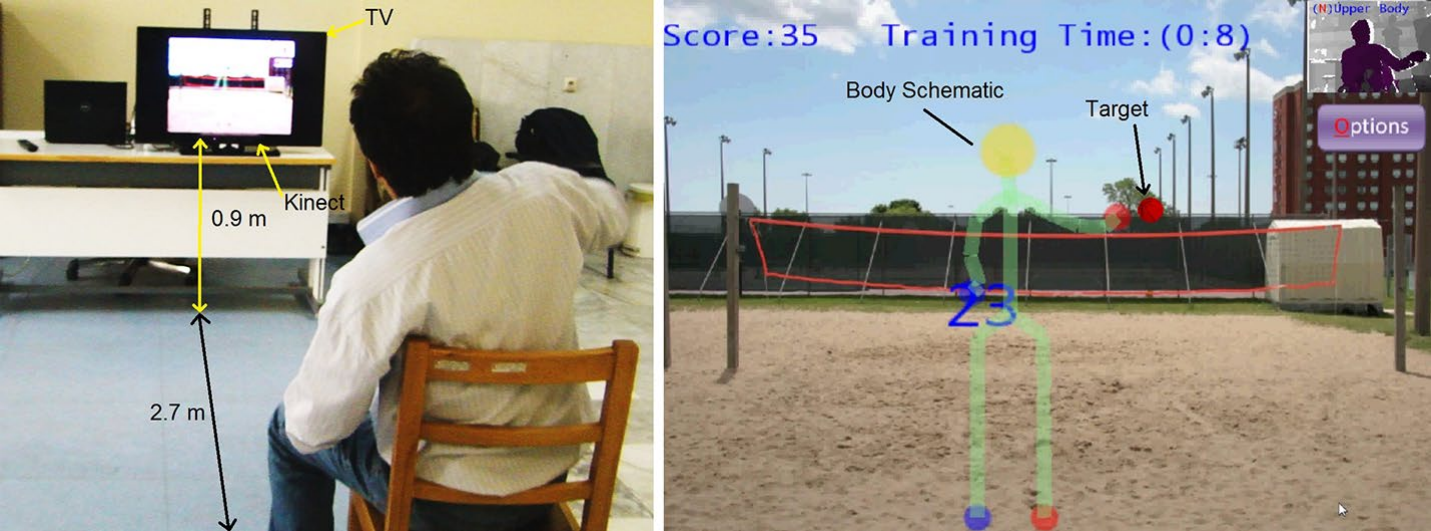
A patient in a test session and data capture program interface.

In the program, they were instructed to move their hands in order to intercept and catch several approaching balls. Balls were sent toward the subject using a predefined pattern shown in Fig. 1. All of the targets are on a plane parallel to the frontal plane but reaching them requires movements in the three dimensional space. The program also provided audio feedback to the patients based on their performance. During the tests, all upper body joints’ positions (Hand, Wrist, Elbow, Shoulder, Shoulder Center, Head and Waist position) were recorded for further analysis using Kinect’s skeleton tracking driver. A picture of one of the patients in a test session and the data capture program interface is shown in Fig. 2. Data capture program guided the patient through training exercises. In this figure a red ball is approaching the patient which implies that the patient should intercept the ball using his red (right) hand.

### The protocol of reliability assessment

The intra-session and inter-session variabilities were adopted for this study, where the former concerns the same session and the latter deals with day-by-day reliability of the system.

Each test session was divided into 4 sub-sessions which lasted 5 min each with 2-min rest intervals. In the test period, subjects were told to catch as many balls as they could. The tests were held twice a week for the patients. They were asked to continue this routine for at least 4 weeks. Therefore, each patient had a minimum of 8 sessions and 32 sub-sessions of training. In order to lower the effects of any misunderstandings about the instructions and adaptation to the virtual environment of the tests, the first test session was considered as an orientation. Therefore, the intra-session reliability analysis was applied to performance indices from the second session.

The inter-session reliability approach was applied to the second sub-session of the last two sessions of each patient’s tests. This choice was to lower the systematic error related to the patient’s progress as its slope leveled off with time. It should be noted that in this study, all patients did their conventional physical therapy sessions two or three times a week and these tests took place parallel with their usual rehabilitation program.

### Performance indices measurement

The positions of upper body joint centers were recorded by Kinect at a sampling frequency of about 30 Hz during the tests. The sampling frequency of Kinect fluctuated between 25.61 and 34.72 Hz with a mean frequency of 29.9 Hz and a standard deviation of 2.67 Hz.

In order to extract performance indices, hand velocity, acceleration and jerk had to be calculated. In these calculations, Kinect’s reference frame was used as the main coordinate system. Since numerical derivation to find velocity and acceleration intensifies the noise, it was essential to smoothen the data before any derivation. For this purpose, a B-spline (which is a piecewise polynomial function of order *k* [30]) was fitted to the position data. The order of B-spline used in this study was 6. The output of this method was a smooth function which was differentiable up to 5 times.

Subsequently, indices of movement performance for each hand, were extracted and calculated as listed below:

1. Mean velocity (*MV*): the mean value of the hand velocity [15] is defined as
  1 $$MV = \frac{{\mathop \sum \nolimits_{i = 1}^{N} V_{i} }}{N}$$
  where $V_{i}$ is the hand velocity at the *i*th sample of data, and *N* is the number of data samples.
2. Normalized mean speed (*NMS*): it is the mean value of the hand velocity divided by its maximum value [15],
  2 $$NMS = \frac{MV}{{V_{max} }}$$
3. Normalized speed peaks (*NSP*): speed peaks are points where acceleration trajectory crosses the *x*-axis. *NSP* is defined as the number of speed peaks divided by the number of data samples [15],
  3 $$NSP = \frac{\text{Number of speed peaks}}{N}$$
4. Logarithm of dimensionless jerk (*LJ*): it is the logarithm of median of hand’s dimensionless jerk [19],
  4 $$LJ = {\text{Log}}\,\left({\text{median}}\left(\frac{(\mathop \smallint _{{t_{1} }}^{{t_{2} }} (\dddot X^{2} + \dddot Y^{2} + \dddot Z^{2} )dt)(t_{2} - t_{1} )}{V_{mean}^{2}} \right) \right)$$
  where $t_{1}$ is the start time and $t_{2}$ is the end time of the movement, *X*, *Y* and *Z* are the positions of the hand measured by Kinect, and $V_{mean}$ is the mean velocity in the movement.
5. Curvature (*C*): it is the logarithm of the median of hand’s path curvature [17],
  5 $$C = {\text{Log}}\,\left({\text{median}}\left(\sqrt{\frac{\mathop(\dot X^{2} + \dot Y^{2} + \dot Z^{2})(\ddot X^{2} + \ddot Y^{2} + \ddot Z^{2})-(\dot X\ddot X+\dot Y\ddot Y+\dot Z\ddot Z)^{2}}{(\dot X^{2} + \dot Y^{2} + \dot Z^{2})^{3}}}\right) \right)$$
  where *X, Y* and *Z* are positions of the hand measured by Kinect.
6. Spectral arc length (*SAL*): it is the negative arc length of the frequency-normalized Fourier magnitude spectrum of the speed profile [18],
  6 $$SAL = - \mathop \int \limits_{0}^{{\omega_{c} }} \sqrt {\left( {\frac{1}{{\omega_{c} }}} \right)^{2} + \left( {\frac{{d\hat{V}\left( \omega \right)}}{d\omega }} \right)^{2} } d\omega , \hat{V}\left( \omega \right) = \frac{V(\omega )}{V(0)}x$$
  where $V(\omega )$ is the Fourier magnitude spectrum of $V(t)$, and $\omega_{c}$ is the frequency band occupied by the given movement ($\omega_{c} = 40\pi{\text{}}\ rad/s$).
7. Shoulder angle with body (*SA*): the mean value of arm angle with body,
8. Elbow angle (*EA*): the mean value of elbow angle.

Both patients and healthy subjects did the exercises following the program guidance. All of the performance indices were measured in every reaching movement and their overall average in each sub-session was calculated so as to obtain one value for each training sub-session.

### Statistical analysis

This study has followed the statistical methods used by Colombo et al. [31] who assessed performance indices reliability measured by robotic systems.

To reach a general view of the variability of the measured performance indices, a scatter plot was drawn for each of the indices measured over two sub-sessions of a single session and repeated measures ANOVA (analysis of variance) was used to calculate the reliability [32, 33].

Naturally, tests such as those carried out in this study involve a parameter of learning, and therefore, existence of learning-related error is taken for granted and not by any means any flaw of the tests. Nevertheless, to reduce the effects of this error on the reliability of intra-session indices, the first sub-session of each test was overlooked to let the patients adapt to the circumstances of the test, and the last 3 sub-sessions were investigated. As for the inter-session reliability of the tests, the last two sessions were analyzed to lower the influence of the learning procedure.

To model the system, *N* subjects with *M* repeated measurements of continuous variable *P* were considered. A mathematical model for measurements of *P* was considered here as:

7 $$P_{ij} = T + t_{i} + S_{ij} + R_{ij}$$

where $P_{ij}$ is the $j$th measurement ($j = 1, \ldots ,M$) made on the *i*th subject ($i = 1, \ldots ,N$), $T$ is the true value of the variable, $t_{i}$ is the subject’s effects on the true value, $S_{ij}$ is the systematic error and $R_{ij}$ is the random error. $t_{i} ,{\text{}}\ S_{ij}$, and $R_{ij}$ are independent random errors which are normally distributed with means of 0 and variances of $\sigma_{t}^{2} ,{\text{}}\ \sigma_{S}^{2}$, and $\sigma_{R}^{2}$, respectively [31, 34, 35]. The reliability of parameter *P* can be calculated using intra-class correlation coefficient (*ICC*) defined as [35]:

8 $${\text{Reliability = }}\frac{\text{Between subjects variability}}{{{\text{Between subjects variability}}\,{ + }\,{\text{error}}}}$$

9 $$R_{u} = \frac{{\sigma_{t}^{2} }}{{\sigma_{t}^{2} + \sigma_{S}^{2} + \sigma_{R}^{2} }}$$

Since this study did not consider the systematic error, Eq. (9) was reduced to Eq. (10):

10 $$R = \frac{{\sigma_{t}^{2} }}{{\sigma_{t}^{2} + \sigma_{R}^{2} }}$$

Both inter-session and intra-session reliabilities were calculated as positive values between 0 and 1. A model of repeated-measures ANOVA was used to determine this parameter. In this method $\sigma_{t}$ and $\sigma_{r}$ were determined as:

11 $$\sigma_{t} = \frac{{MS_{S} - MS_{E} }}{k}$$

12 $$\sigma_{R} = MS_{E}$$

where $MS_{S}$ is the subjects difference mean square and calculated based on differences among subjects in measurements of each trial, $MS_{E}$ is error mean square which was calculated based on the difference between evaluations of one subject’s trials and *k* is the number of trials which is 2 for this study.

By replacing Eqs. (11) and (12) in Eq. (10), McGraw and Wong’s [33] 2-way fixed model equation (*C*, 1) was obtained as below:

13 $$R = \frac{{{\text{MS}}_{S} - {\text{MS}}_{E} }}{{{\text{MS}}_{S} + \left( {k - 1} \right){\text{MS}}_{E} }}$$

Moreover, the standard error of measurement (*SEM*) was calculated which makes an absolute index of reliability available, and allows for the quantification of each measurement’s precision. *SEM* was defined as the square root of the mean square from ANOVA results. *SEM* has the same units of each measured indices and encompasses components of random and systematic error of measurement.

14 $${\text{SEM}} = \sqrt {{\text{MS}}_{E} }$$

Furthermore, *SEM*’s coefficient of variation (*CV*) was calculated in order to optimize the result comparison in cases of various unites and scales [36]. The *CV* of *SEM* was defined as the ratio between *SEM* and each index’s overall mean and was presented as a percentage. Another parameter, calculated in this study, was the minimal detectable difference (*MDD* [33]) which indicates the minimum difference required to state a significant change in an index. Equation (15) demonstrates how *MDD* was calculated [33]. This parameter enables the examiner to realize whether any noticeable change has occurred in the movement quality index.

15 $${\text{MDD}} = {\text{SEM}} \times 1.96 \times \sqrt 2$$

## Results

**Fig. 3:**
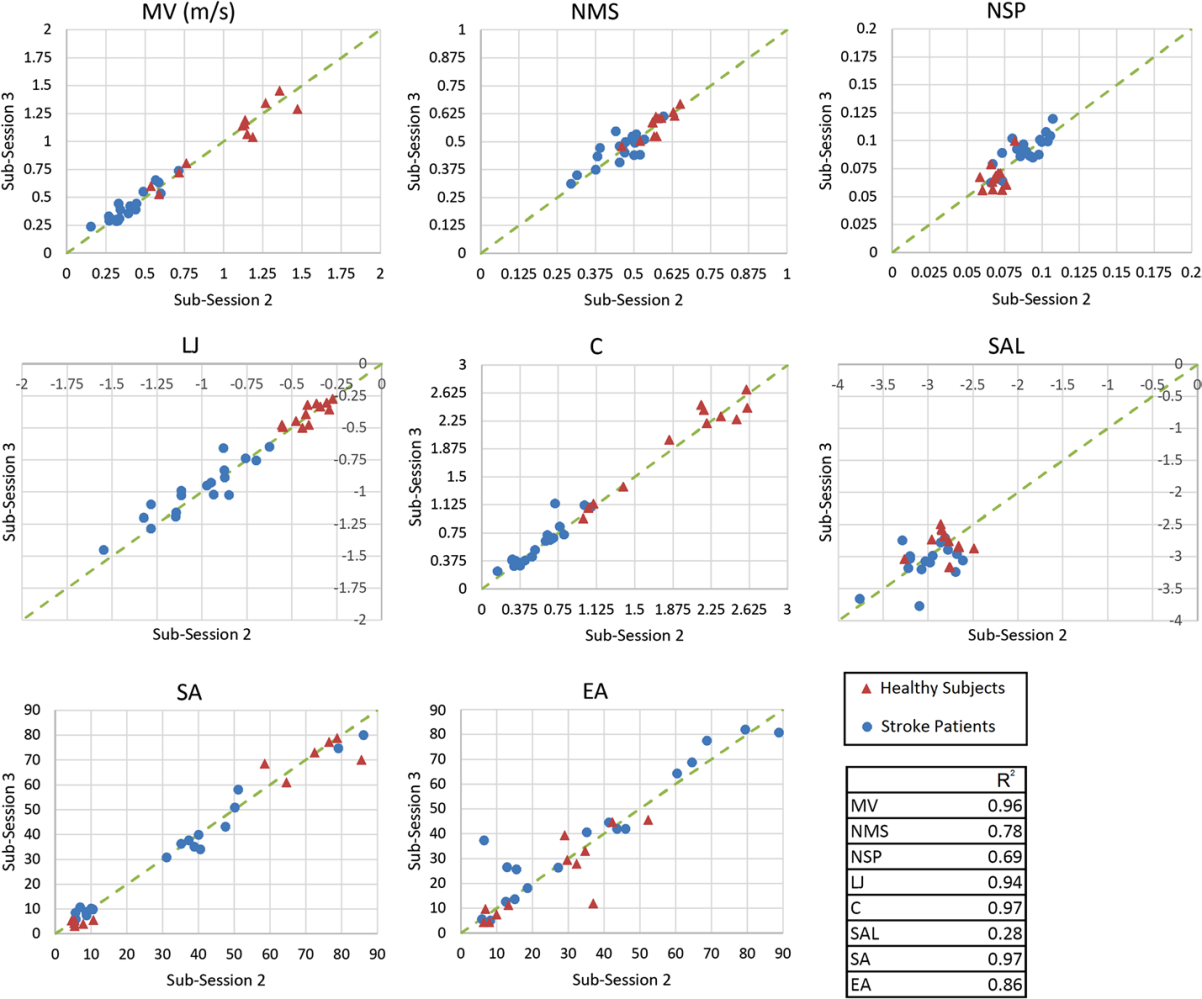
Mean value of each performance index measured in sub-session 2 vs. sub-session 3 of the second test session in healthy subjects (*triangles*) and stroke patients (*circles*) with data coefficient of determination related to identity line.

As described in the last section, to obtain an overview of the results, the scatter plot of each performance index was plotted for two sub-sessions of the second test session as shown in Fig. 3. Each part of this figure reports the mean value of one index measured in sub-session 2 vs. sub-session 3 for all patients and healthy subjects. Since the indices were measured in two consecutive sub-sessions, consistency of the index required the data points to be located close to the identity line. To compare performance indices, coefficient of determination (*R*^2^) [37] was found with respect to the identity line and shown in Fig. 3. The figure shows that, except *SAL*, the other indices are very close to the identity line, implying better consistencies. Among these indices, *MV*, *LJ*, *C* and *SA* show the best performance with *R*^2^ greater than 0.9. This figure also demonstrates a good separation between healthy subjects and patients in *MV*, *NMS*, *NSP*, *LJ* and *C*. This means that these indices are better nominees for assessing the patient’s state and progress. Also, the values of *MV*, *LJ* and *C* are separated more widely along the identity line which may imply a better resolution in distinguishing between the patients’ status.

**Fig. 4:**
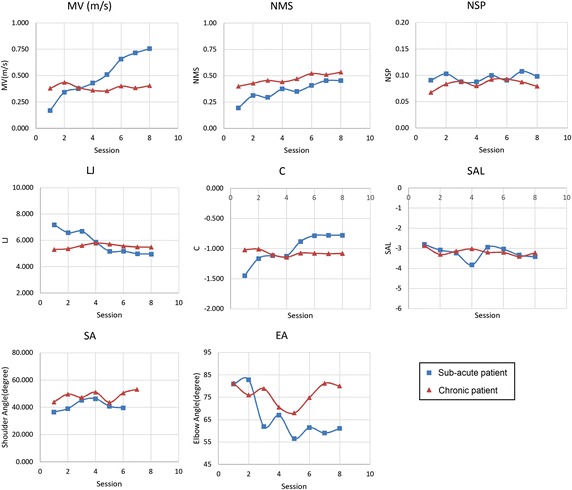
Motion performance indices for one sub-acute and one chronic patient.

Motion performance indices for one sub-acute and one chronic patient in 8 sessions are shown in Fig. 4. In this figure, the mean value of each performance index in the four sub-sessions is considered as the value for that session. As shown in this figure, for the sub-acute patient *MV*, *NMS*, *LJ* and *C* change uniformly in the period of the test which indicates patient’s progress in this span of time. On the other hand, for the chronic patient, the variation of the indices is not significant which is because of his lower rate of recovery relative to the sub-acute patient.

The mean value, standard deviation, and intra-session test–retest reliability of the results measured by Kinect from healthy subjects and stroke patients are presented in Tables 1 and 2. The intra-session test–retest reliability of the results is expressed by *ICC*, *SEM*, *CV* and *MDD* as defined in the last section. These tables show high *ICC* values (more than 0.8) for all indices except *NSP* and *SAL* for both patients and healthy subjects. The *CV* in healthy subjects is mostly lower than stroke patients which points to the higher consistency of healthy subjects’ movements. The worst performance of *SEM* is for *EA* which has more than 15 % of *CV* for both patients and healthy subjects. A comparison of the *MDD* and the difference between mean values of indices for healthy subjects and stroke patients indicates that there is a meaningful difference between these numbers. This comparison shows that one can distinguish patients from healthy subjects using most of these indices.

**Table 1:** Intra-session reliability parameters in healthy subjects

Performance Indices	Mean ± SD	*ICC*	*SEM*	*CV*	*MDD*
*MV* (m/s)	1.033 ± 0.296	0.96	0.083	8.02	0.230
*NMS*	0.576 ± 0.053	0.86	0.024	4.19	0.067
*NSP*	0.068 ± 0.009	0.42	0.006	9.35	0.018
*LJ*	−0.399 ± 0.087	0.81	0.034	8.44	0.093
*C* (m)	1.923 ± 0.609	0.97	0.114	5.94	0.316
*SAL*	−2.812 ± 0.202	0.34	0.116	4.12	0.322
*SA* (°)	38.86 ± 34.45	0.98	2.216	5.70	6.144
*EA* (°)	23.75 ± 15.51	0.86	4.371	18.4	12.11

**Table 2:** Intra-session reliability parameters in stroke patients

Performance indices	Mean ± SD	*ICC*	*SEM*	*CV*	*MDD*
*MV* (m/s)	0.415 ± 0.141	0.93	0.057	13.8	0.159
*NMS*	0.459 ± 0.075	0.81	0.045	9.77	0.124
*NSP*	0.090 ± 0.014	0.77	0.007	8.33	0.021
*LJ*	−1.001 ± 0.228	0.91	0.087	8.68	0.242
*C* (m)	0.588 ± 0.269	0.91	0.07	11.9	0.194
*SAL*	−3.136 ± 0.385	0.52	0.31	9.85	0.856
*SA* (°)	32.69 ± 23.64	0.99	4.14	12.7	11.53
*EA* (°)	37.86 ± 25.92	0.94	5.84	15.4	16.19

The mean value, standard deviation and inter-session test–retest reliability of the results measured by Kinect from stroke patients are presented in Table 3. This table, also, shows high *ICC* values (more than 0.9) for all indices except *NSP*, *NMS* and *SAL*. In this table, similar to the others, the worst performance of *SEM* belongs to *EA* which has more than 16 % of *CV*. Comparing mean values of indices in this table and Tables 1 and 2 shows that mean values of indices from Table 3 lie between those from Tables 1 and 2. For a better comparison, the mean value and standard deviation of the differences between indices from the second session and the last session of therapy are shown in Table 4. As reported in Table 4, only in *MV*, *LJ* and *C*, the mean difference is more than *MDD*. This shows that only these three indices present a meaningful variation in 1 month of therapy. Thus using these indices, one is able to measure patients’ progress in this period.

**Table 3:** Inter-session reliability parameters in stroke patients

Performance indices	Mean ± SD	*ICC*	*SEM*	*CV*	*MDD*
*MV* (m/s)	0.669 ± 0.336	0.94	0.086	12.8	0.239
*NMS*	0.459 ± 0.074	0.6	0.034	7.36	0.093
*NSP*	0.085 ± 0.012	0.71	0.007	7.9	0.019
*LJ*	−0.779 ± 0.293	0.95	0.089	11.4	0.247
*C* (m)	0.951 ± 0.517	0.96	0.141	14.7	0.389
*SAL*	−3.19 ± 0.996	0.12	0.413	12.9	1.144
*SA* (°)	30.32 ± 24.46	0.96	4.22	13.9	11.71
*EA* (°)	33.12 ± 19.33	0.92	5.49	16.58	15.22

**Table 4:** Change of indices in stroke patients after 1 month of rehabilitation

Performance indices	Change (mean ± SD)	*MDD*
*MV* (m/s)	0.292 ± 0.303	0.239
*NMS*	0.001 ± 0.126	0.093
*NSP*	−0.005 ± 0.021	0.019
*LJ*	0.285 ± 0.222	0.247
*C* (m)	0.443 ± 0.399	0.389
*SAL*	−0.058 ± 1.061	1.144
*SA* (°)	−8.52 ± 18.19	11.71
*EA* (°)	−8.71 ± 15.61	15.22

## Discussions

The results, as summarized in Tables 1, 2, 3, 4, indicate an acceptable intra-session and inter-session reliability for measuring *MV*, *LJ*, *C*, *SA* and *EA*. They are comparable to those computed with robotic systems [31] or clinical assessment scales reported in the literature [38, 39]. The measured *ICC* of Kinect and robotic devices reported by Colombo et al. [31] are summarized in Table 5. As shown in the table, the *ICC* values of *MV* are quite close. Although, the *ICC* of *LJ*, *C*, *SA* and *EA* are not reported in the Colombo’s work, they have values close to the *ICC* of *MV* when measured by Kinect. The *ICC* of the other indices, i.e. *NMS*, *NSP* and *SAL*, are relatively lower when measured by Kinect and hence not as reliable as the other indices.

**Table 5:** Measured *ICC* of Kinect and Colombo et al. [31] results for robotic devices

Performance indices	Healthy *ICC*	Patients *ICC*		Healthy *ICC* [31]	Patients *ICC* [31]	
	I1^a^	I1	I2^b^	I1	I1	I2
*MV* (m/s)	0.96	0.93	0.94	0.95	0.97	0.93
*NMS*	0.86	0.81	0.6	0.99	0.94	0.91
*NSP*	0.42	0.77	0.71	0.85	0.82	0.95
*LJ*	0.81	0.91	0.95	–	–	–
*C* (m)	0.97	0.91	0.96	–	–	–
*SAL*	0.34	0.52	0.12	0.92	0.83	0.95
*SA* (°)	0.98	0.99	0.96	–	–	–
*EA* (°)	0.86	0.94	0.92	–	–	–

^a^Intra session reliability.

^b^Inter session reliability.

Comparing the results of Tables 1 and 2 shows that there are significant differences between the mean values of all indices for healthy subjects and stroke patients. Also, all of the differences are larger than *MDD*, pointing to the fact that all of these indices while measured by Kinect can be used in separating patients from healthy subjects. However, comparison between the results of Tables 2 and 3 which is summarized in Table 4 implies that only the variations of *MV*, *LJ* and *C* are significant after 1 month of therapy and the other indices are not capable of reflecting patients’ progress in this period of time.

A comparison of the results of Tables 3 and 1 shows that even after 1 month of therapy, there is a significant difference between all mean values of the indices for healthy subjects and stroke patients. This means that even after 1 month of therapy, stroke patients and healthy subjects can be easily separated by all of the indices although none of *NMS*, *NSP*, *SAL*, *SA* and *EA* are sufficiently sensitive for assessing patients’ progress in this period of rehabilitation. Also, this indicates that saturation doesn’t happen when using *MV*, *LJ* and *C* as patients’ progress indicators. After 1 month of therapy, they still maintain a considerable distance from a healthy subject.

As stated above, *NMS*, *NSP*, *SAL*, *SA* and *EA* seem to be incapable of reflecting the patient’s progress when measured by Kinect. This is in conflict with a number of other related studies [15, 18, 31] that show significant changes in these indices during 1–6 months of rehabilitation. This has to be due to Kinect’s lower accuracy in measuring those particular indices which could lower their sensitivity. In related literatures, clinical measurement systems were used which are more accurate than Kinect [25, 40–42].

The inter-session and intra-session test–retest reliability of indices measured by Kinect for healthy subjects had lower than 4% difference. Due to this small difference, the inter-session test–retest reliability results are not reported for the sake of brevity.

As Fig. 4 demonstrates, the learning effect on most of the performance indices of sub-acute patients are significant in the first month of therapy. In later studies, it is suggested to prolong the test time to 6 months in order to lower the effects of patient’s learning on inter-session reliability.

Finally it should be remembered that the objective of this study was to investigate the reliability of performance indices when measured by Kinect. In future works, it is necessary to study the measurement accuracy of these indices in comparison to popular clinical devices such as marker-based optical and inertial ones. Furthermore, determination of the correlations between the performance indices and the clinical measures of the subject’s quality of motion is another interesting subject which is being pursued by the authors in a separate study.

## Conclusions

In this study, a systematic approach was followed to assess the intra-session and inter-session reliability of performance indices measured by Microsoft Kinect. The results of this study showed that, among the performance indices, *MV*, *LJ*, *C*, *SA* and *EA* had more than 0.9 *ICC* together with an acceptable *SEM* and *CV* in both stroke patients and healthy subjects across sessions. However, only *MV*, *LJ* and *C* showed significant variations after 1 month of therapy and hence concluded to be suitable for progress evaluation of patients. The results are promising for the development of home-based rehabilitation systems using Kinect as an affordable motion capture sensor.
